# Evaluation of a Course to Teach Medical Students Latent Hazard Identification in the Operating Room

**DOI:** 10.7759/cureus.56367

**Published:** 2024-03-18

**Authors:** Natasha C Cammer, Kristen M Mascarenhas, Marianfeli C Delgado-Landino, Danielle B Horn, Roxanna J Araya, Richard H Epstein, Jean R Corvington, Catherine P Marudo, Alecia L Stein, Joni M Maga

**Affiliations:** 1 Anesthesiology, Perioperative Medicine, and Pain Management, University of Miami Miller School of Medicine, Miami, USA; 2 Center for Patient Safety, University of Miami/Jackson Memorial Hospital, Jackson Health System, Miami, USA

**Keywords:** teaching, situational awareness, simulation training, patient safety, operating rooms, medical students

## Abstract

Introduction: To improve situational awareness in the operating room (OR), a virtual online operating room of hazards (ROH) with deliberately placed risks was created. We hypothesized that subjects first participating in the virtual online ROH would identify more hazards during an in-person ROH exercise in a physical OR than those in the control group who only received didactic training.

Methods: We conducted a randomized controlled trial at a major academic medical center, enrolling 48 pre-clinical medical students with no previous OR exposure during their classes. Control and experimental group subjects participated in a brief, online didactic orientation session conducted live over Zoom (Zoom Video Communications, Inc., San Jose, CA) to learn about latent hazards in the OR. Experimental group subjects further interacted with a virtual online operating ROH in which latent hazards were present. The fraction of deliberately created latent hazards placed in a physical, in-person OR identified by subjects was calculated.

Results: Experimental group subjects identified a significantly larger fraction of the created hazards (41.3%) than the control group (difference = 16.4%, 95% CI: 11.3% to 21.4%, P < 0.0001). There was no difference in the number of non-hazards misidentified as hazards between the groups.

Conclusions: Participation in the virtual online environment resulted in greater recognition of latent operating room hazards during a simulation conducted in a physical, in-person OR than in a didactic experience alone. Because creating an in-room experience to teach the identification of latent hazards in an OR is resource-intensive and requires removing the OR from clinical use, we recommend the virtual online approach described for training purposes. Adding items most misidentified as hazards is suggested for future implementation.

## Introduction

Situational awareness, defined as “conscious knowledge of the immediate environment and the events that are occurring in it” [1], has the potential to improve health outcomes [2,3]. As situational awareness is a trainable skill [4-6], many institutions offer programs using different models to enhance it, aiming to improve patient safety and subsequent health outcomes. Although many viable approaches to such training in the hospital environment exist, training on virtual platforms maximizes accessibility and efficiency.

We previously conducted a trial of a three-dimensional (3D) virtual tour activity inspired by the “Room of Horrors,” which originated as an in-person activity during which students identify hazards in a simulated hospital room. Our adaptation transformed this activity into a virtual platform highlighting patient safety threats in a standard hospital room [7]. Results from that study showed subjects were comfortable navigating the 3D virtual simulation platform and had improved hazard identification in a hospital room after completing the activity. For the current study, we used the same technology to create a virtual representation of an operating room (OR) to similarly teach students to recognize latent hazards in this more complex environment.

The OR is a highly complex setting where patient safety hazards can result in life-altering consequences, making training essential for OR healthcare professionals. In a study using virtual reality simulation to train situational awareness in the OR, Bracq et al. outlined challenges such as environmental factors like noise and time pressure, individual factors like fatigue and stress, and cognitive factors like information overload and attentional capacity primarily through lengthy procedures [8]. Previous studies have suggested that training to target these domains specifically can improve situational awareness in the OR and overall teamwork and clinical practice [9,10].

With these challenges in mind, the OR was the ideal setting for us to target our next stage of virtual patient safety training. We hypothesized that participation in an online virtual room of hazards (ROH) experience would result in the identification of a higher percentage of deliberately created hazards in a physical OR with a simulated patient than only participation in a didactic presentation.

## Materials and methods

Institutional review board exemption for documentation of consent from the University of Miami was obtained, with subject deidentification specified (IRB#20230789, dated August 4, 2023). In addition, because the study was carried out at Jackson Memorial Hospital, the Jackson Health System Office of Research also provided approval per institutional requirements (letter dated August 8, 2023). All pre-clinical medical students from the University of Miami Miller School of Medicine were invited to participate in person, immediately prior to a scheduled didactic. Only active first or second-year medical students were included; subjects who were unable to commit to attending all study activities were excluded. The 61 individuals who verbally consented were randomized to either the control or experimental group using a lookup table generated by the rand function in Excel (Microsoft, Redmond, WA).

**Figure 1:**
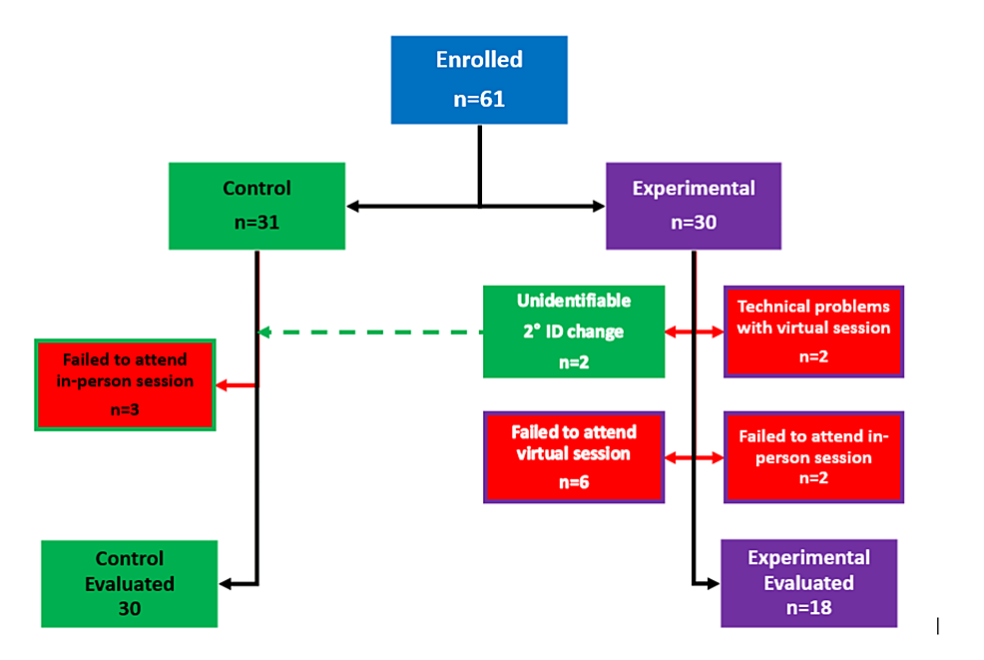
Flow diagram of study enrollment The dotted green line between the experimental and control groups represents two subjects who changed their anonymous identifier between the virtual and the in-person simulation. See the Methods for a detailed discussion of the rationale for moving them to the control group and the potential bias introduced.

The study protocol included an educational presentation for all subjects (day one), a virtual ROH simulation for the experimental group subjects (day four), and an in-person ROH simulation in a physical OR for all subjects (day eight to 11). To maintain participants' anonymity and accurately track their responses across simulation activities, each subject generated their own anonymized identifier using a hash-based message authentication code generated from the Duo Mobile authentication application (Cisco, San Jose, CA). They provided this identifier when participating in the simulation activities. Subjects in the experimental group were instructed to retain this six-digit code and provide it for the virtual and in-person simulations. Unfortunately, two subjects in the experimental group changed their identifier for the in-person scenario. This was detected during analysis, as 20 subjects from the experimental group completed the in-person simulation, but only 18 of the codes matched those present for the virtual simulation. These two experimental subjects’ anonymous identifiers could not be distinguished from the control subjects’ identifiers. Because we could not re-identify them without violating the protocol submitted to the IRB, these two experimental group subjects were included in the statistical analysis as control group subjects. The consequence of this created bias in the direction of showing no benefit from the experimental intervention (i.e., toward rejection of the hypothesis that the online simulation would improve latent hazard recognition). Thus, the presence of a statistically significant difference favoring the experimental group would be more reliable than had the two experimental subjects not changed their identifier (Figure 1).

Educational presentation

A 15-minute educational presentation via Zoom (Zoom Video Communications, Inc., San Jose, CA) oriented the subjects to the study design, learning objectives, operating room layout, common OR hazards, definitions such as situational awareness, and information about the OR and standard equipment. Subjects who did not participate in the subsequent in-person simulation activity were excluded.

Creating the virtual and in-person ROH

Hazards were chosen based on a literature review of the most frequently reported hazards in ORs and from clinical insights of the anesthesiologists on our research team [11,12]. The MeSH terms used for the literature search included “anesthesiology; medication safety; operating room; simulation training; medication errors; patient safety.” These identified hazards were categorized based on patient characteristics, interactions with the anesthesiologist, medication labeling, communication, environmental factors, and supplies.

Luxhunters Productions Inc. (Miami, FL), a local company that creates virtual housing walkthroughs for real estate agents, was contracted to produce a 3D virtual tour of an operating room that was closed for patient care during the day of filming. Filming focused on highlighting 52 commonly encountered hazards (a full list is provided in Appendix A titled "Virtual operating room of hazards"). An interactive video was created using a spatial data software package (Matterport Inc., Sunnyvale, CA). This web-based platform created a 3D digital portrayal of our ROH. Access to the virtual tour required a device with internet access, such as a tablet, laptop, or desktop computer. After filming, all hazards were removed, and the OR was returned to its previous state. The research team met with the Luxhunters representative to edit the virtual ROH module; then the final simulation was made accessible via a web link (https://bit.ly/OR_ROH). The cost of producing the virtual activity was US$560.

An in-person ROH with 39 hazards within the same categories but not identical to those presented in the virtual ROH was designed (e.g., mislabeled syringes had different errors than those presented online). See the full list in Appendix B titled "In-person operating room of hazards." A fully equipped obstetrics OR, closed for patient care during the four days of the activity, was made available by the operating room administrators at Jackson Memorial Hospital. The ROH contained standard OR equipment and supplies. A full-bodied mannequin with a simulated peripheral IV, Foley catheter, forced air warming blanket, and wristband (with fictitious identifiers) undergoing a simulated surgical procedure was positioned on the OR table and connected to the anesthesia machine breathing circuit. A research team member acted as the simulated anesthesiologist in the room.

The 3D virtual experience

Experimental group subjects underwent a five-minute briefing on the study's objectives and were given instructions for the activity. They were asked to enter the virtual ROH and document all hazards identified on a Qualtrics^XM ^(Qualtrics, Inc., Provo, UT) form, a customizable survey collection software tool, within 10 minutes as they explored the room.

**Figure 2:**
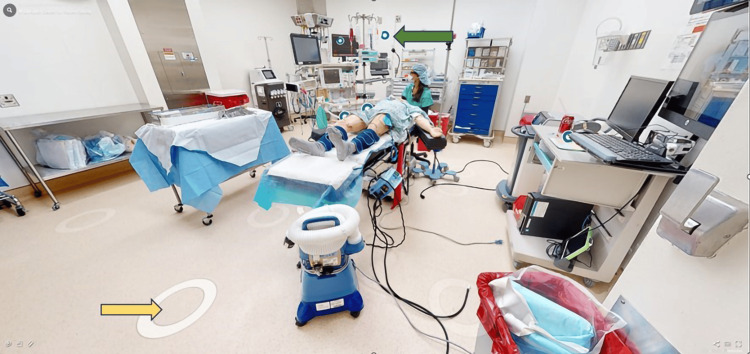
Virtual operating room of hazards The yellow arrow indicates circles used for navigating throughout the room, while the green arrow indicates an example of a tag that can be clicked to display embedded photos. For a higher-resolution view, visit the link to the online activity (https://bit.ly/OR_ROH).

**Figure 3:**
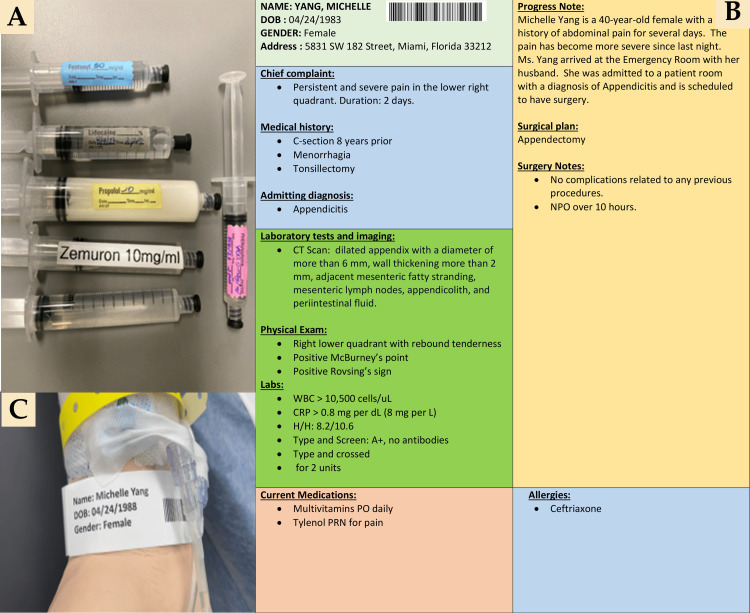
Virtual operating room tags Photos illustrate a close-up view of medication labels on the syringes (A), the patient’s chart (B), and the patient’s identification wristband (C). For a higher-resolution view, visit the link to the online activity (https://bit.ly/OR_ROH). Please note this is a fictitious patient, chart, and fictitious protected health information for the purposes of simulation.

The subjects clicked on the link to initiate the module, which opened with an aerial dollhouse view of the entire OR and exterior area, gliding the user inside the OR to the foot of the patient bed. To navigate through the virtual room, the subjects used their device’s pointing tool to select white ellipses located on the floor of the OR. Six teal circles with a white center were provided (Figure 2). They displayed an expanded view of relevant items when hovering over or clicking the circle, i.e., medication labels on the syringes (Figure 3A), the patient’s chart (with fictitious identifiers) (Figure 3B), and the patient’s identification wristband (with fictitious identifiers) (Figure 3C). Refer to the online link (https://bit.ly/OR_ROH) for expanded visualization. After completing the virtual ROH session, a research team member conducted a 10-minute group debriefing with all the subjects who participated.

In-person OR experience

Control group subjects underwent a five-minute briefing on the study's objectives and were given instructions for the activity. All subjects were provided individual appointment times to participate in the in-person ROH, and a researcher not involved in the simulation took attendance by recording the subjects’ anonymized identifiers. To comply with OR sterility guidelines, subjects donned a surgical scrub cap, a mask, and a surgical one-piece coverall. Each subject was allowed 10 minutes to inspect the OR while documenting identified hazards in Qualtrics using either their own or a provided handheld device. Following the in-person ROH activity, an individualized debriefing was conducted.

Data recording

After all data were collected from the online, virtual simulation (experimental group), one researcher coded the experimental data, one coded the control data, and a third researcher oversaw coding for consistency. If there was uncertainty in coding an entry, the three researchers reached a consensus judgment. Each entry was coded as having identified an intended hazard, mistakenly identified as non-hazard, or as erroneous. Hazards that were present but not identified were coded as having been missed. Mistakenly identified hazards were defined as those not created from the curated list. See the full list titled "Mistakenly identified hazards from the in-person room of hazards" in Appendix C. Erroneous hazards were statements that the research team could not cross-reference with the activity or result from unclear spelling or wording errors, i.e., “seems elevated,” and “too many tolls [sic] are out.” For the virtual session, each subject in the experimental group was scored based on the number of hazards correctly identified divided by 52 (the total number of hazards in the virtual room). Mistakenly identified hazards and erroneous hazards were analyzed separately.

For the in-person session in the physical OR, the data were recorded and classified in the same manner as described above. In both types of simulations, each hazard was grouped into its respective subcategory (see Appendices A-C).

Statistical analysis

Based on an alpha of 0.05 and 80% power, a minimum of 12 subjects per group was required to be able to detect a 12% improvement in the fraction of hazards identified by the experimental group using a one-sided, two-sample means test, an estimated standard deviation between 0.1 and 0.2, and Satterthwaite’s correction for unequal variance. Additional subjects were enrolled to account for anticipated dropouts. The primary analysis assessed the scores for the control versus the experimental group in identifying actual OR hazards during the in-person experience. The in-person experience score was calculated by dividing the number of hazards identified by subjects by 39 (the total number of hazards present in the OR). An unpaired, one-sided t-test was used to test if the performance of the experimental group was superior to that of the control group. A one-sided test was applied based on the expectation that the virtual training would improve performance, as was found in the previous study [7]. Further analysis was performed using an unpaired, two-sided t-test to determine whether the mean number of mistakenly identified hazards was statistically different between the experimental and control groups. A two-sided test was applied because there was no expectation that training would reduce the incidence of erroneous identification of hazards. We addressed the sample size and variance differences between the two groups based on the recommendations by Rusticus and Lovato [13]. We performed a two one-sided test (TOST) with Welch’s approximation for the hypothesis that the absolute value of the difference between the two groups would be ≥10%. For all tests, P < 0.05 was required to claim statistical significance.

Qualitative feedback

Following the virtual and in-person debriefing sessions, subjects completed de-identified programmatic evaluations using five-point Likert scales and free text. Subjects recorded agreement with the provided statements on a scale from strongly disagree (1) to strongly agree (5). Data were analyzed to determine the number and percentage of participants agreeing with each statement to evaluate participants' satisfaction. In the free-text response section of the questionnaire, subjects were asked to provide feedback regarding their experience and to suggest improvements that we could use to optimize this activity.

## Results

Subject enrollment and dropouts

A total of 61 pre-clinical first- and second-year medical students enrolled in this study, none of whom had any formal exposure to the operating room during their previous medical school classes. Thirty subjects were randomized to the experimental group and 31 to the control group, all of whom attended the initial educational session. There were three dropouts from the control group and 10 from the experimental group. As described in the Methods, two of the experimental group subjects were analyzed in the control group because of issues with their anonymous identifier. This left 30 evaluable control subjects and 18 experimental subjects (Figure 1). Regarding the sample size difference, the one-sided test of the null hypothesis that the difference in the means (0.25-0.41 = -0.16) was less than 0.1 was rejected (t = 10.04, P < 0.0001). The one-sided test of the null hypothesis that the difference in the means (-0.16) was < -0.1 was not rejected (t = -2.42, P = 0.99). Therefore, the two groups were not equivalent, with the performance of the experimental group being better.

Identification of latent hazards

The subjects in the experimental group recorded a significantly higher fraction of the deliberately introduced latent hazards (41.3%) compared to the subjects in the control group (Table 1).

**Table 1:** Latent hazard identification and mistakenly identified hazards CI: confidence interval; t: two-sample t-test.

Metric	Control (n = 30), mean (95% CI)	Experimental (n = 18), mean (95% CI)	Mean difference (95% CI)	P-value
Latent hazards identified (%)	25.0 (22.0 to 27.9)	41.3 (35.7 to 46.0)	16.4 (11.3 to 21.4)	<0.0001 (t = -6.52)
Mistakenly identified hazards (n)	4.37 (3.48 to 5.26)	5.06 (3.46 to 6.65)	0.69 (-0.94 to 2.32)	0.4 (t = -0.85)

The number of mistakenly identified hazards was not different between the control and experimental groups (Table 1). The most frequently identified mistaken hazards were open sterile equipment (n = 13 subjects), no sterile handles on the OR lights (n = 14 subjects), electrical cords in a non-walkable area (e.g., behind the anesthesia machine) not taped to the floor (n = 12 subjects), gloves hanging from the side of the trash can (n = 13 subjects), and a stain on the floor mistakenly identified as urine (n = 10 subjects).

Virtual subject feedback

Responses to the statements with Likert scales (Table 2) were highly positive, with over 161 (80%) being “strongly agree” or “agree” for nine of 10. The exception was for the statement, “Navigating the virtual operating ROH environment was easy,” which received only nine (48%) of such responses (Table 2).

**Table 2:** Subject feedback from the virtual room of hazards Subjects responded to questions using a five-point Likert scale, where 1 indicates “Strongly disagree,” and 5 indicates “Strongly agree.” The data are represented as total numbers and percentages.

Statement	Strongly disagree	Disagree	Neutral	Agree	Strongly agree
1. The objectives of the virtual operating room of hazards activity were clear to me.	0	0	1	10 (53%)	8 (42%)
2. This educational activity met the identified objectives.	0	0	0	8 (42%)	11 (58%)
3. It was a worthwhile learning experience.	0	0	1	3 (16%)	15 (79%)
4. I feel better prepared to begin my operating room-related clerkships.	0	0	1	10 (53%)	8 (42%)
5. This activity increased my comfort level in navigating a virtual operating room of hazards.	0	0	1	8 (42%)	10 (53%)
6. I can demonstrate my ability to recognize patient safety hazards in an operating room and provide examples.	0	0	2	9 (47%)	8 (42%)
7. It would be valuable to periodically repeat the online experience.	0	1	2	7 (37%)	9 (47%)
8. This would be a worthwhile learning activity to add to the medical school curriculum.	0	0	0	8 (42%)	11 (58%)
9. Accessing the virtual operating room of hazards activity was easy.	0	1	0	11 (58%)	7 (37%)
10. Navigating the virtual operating room of hazards environment was easy.	0	4	6	6 (32%)	3 (16%)

Regarding the overall experience for the activity, 13 comments were coded as “positive,” two as “neutral,” and only one as “negative.” An example of a positive comment was: “This was a great experience and seems like an important part of preparing for clerkships.” Neutral comments were “N/A,” while the one negative comment was “Some of the navigating with the software was a little cumbersome and challenging to pick up all the details.” The most frequent comment regarding areas for improvement was the navigation of the virtual room, with 13 (81%) of respondents suggesting that the navigation tools could be improved. Several subjects wrote that it would have been helpful to have more preparatory training on using the software and time to learn how to navigate it before starting the timer.

In-person subject feedback

Notably, of the 48 (100%) subjects, 11 (23%) agreed and 37 (77%) strongly agreed that this training would be worthwhile to add to the medical school curriculum, and 42 (88%) strongly agreed that the in-person activity was a valuable learning experience (Table 3).

**Table 3:** Subject feedback on the in-person room of hazards Subjects responded to questions using a five-point Likert scale, where 1 indicates “Strongly disagree,” and 5 indicates “Strongly agree.” The data are represented as total numbers and percentages.

Statement	Strongly disagree	Disagree	Neutral	Agree	Strongly agree
1. The objectives of the in-person operating room of hazards activity were clear to me.	0	1	2	16 (33%)	29 (60%)
2. This educational activity met the identified objectives.	0	0	0	14 (29%)	34 (71%)
3. It was a worthwhile learning experience.	0	0	0	6 (13%)	42 (88%)
4. I feel better prepared to begin my operating room-related clerkships.	0	0	3	14 (29%)	31 (65%)
5. This activity increased my comfort level in navigating an operating room environment.	0	0	1	19 (40%)	28 (58%)
6. I can demonstrate my ability to recognize patient safety hazards in an operating room and provide examples.	0	1	3	20 (42%)	24 (50%)
7. This would be a worthwhile learning activity to add to the medical school curriculum.	0	0	0	11 (23%)	37 (77%)
8. Accessing the in-person operating room of hazards activity was easy.	0	0	2	19 (40%)	27 (56%)
9. Navigating the in-person operating room of hazards environment was easy.	0	1	2	19 (40%)	26 (54%)

The feedback regarding subjects’ general experience (Table 3) was positive overall with 28 responses coded as “Positive,” one “Neutral,” and two “Negative.” Positive responses included words such as “fun,” “great experience,” “low time commitment,” and “valuable.” Negative responses suggested that the hazards were too detailed. Some suggestions for improvement were to increase engagement in the original educational presentation, such as adding poll questions. Two subjects wrote that they wished they had more time or a warning of how much time was left. Although subjects were notified that the anesthesiologist was part of the simulation, several subjects wrote that they were unsure whether the acting anesthesiologist intentionally created hazards, for example, by looking at their phone.

## Discussion

The results of this study demonstrate that the virtual ROH is an effective training tool to teach pre-clinical medical students latent OR hazard identification. These findings align with other studies indicating that hazard identification is a trainable skill [5,6,14] and support the utility of using virtual simulation for teaching real-world expertise [15].

Both the virtual and in-person activities received positive feedback from learners, but the virtual ROH required fewer resources and offered enhanced flexibility for learners and teachers, making it a valuable module to formally integrate into the curriculum in preparation for clinical clerkships.

Challenges

For the in-person activity, the largest challenge was securing an empty OR for nearly a week. Other challenges included scheduling logistics between OR availability, participants, and study administrators. These challenges were eliminated after the creation of the virtual experience since the online activity has no accessibility or scheduling constraints. However, due to technological limitations, some subjects experienced technical difficulties while navigating the virtual room that prevented them from completing the activity.

The results of this study may be affected by attrition bias. Compared to the control group, a larger percentage of subjects in the experimental group did not complete the activity (three and 10, respectively). This was attributed to the experimental group having to complete three sessions versus two for the in-person group. All study activities were completed during the medical students' free time, in addition to their full course load.

Limitations

Although all subjects were in the pre-clinical phase of medical school, prior knowledge of hazards in the OR may have varied among subjects. After the study was completed, we learned that a few subjects had been previously exposed to the hospital room virtual simulation training created by our group [7]; however, due to blinding, we did not know to which group they were assigned. There was some overlap in the latent hazards presented (e.g., patient identification label errors), so this exposure may have improved their performance during the physical OR simulation activity.

Many students mentioned experiencing technical difficulties while navigating the virtual room. These technical difficulties were due to limitations with the technology itself, especially as the technology was originally designed to display real estate rather than host a simulation activity. In reviewing feedback, students provided many useful suggestions to address this issue, such as a more extensive orientation to introduce the navigation tools prior to beginning the virtual ROH activity, which we plan to implement in future iterations of the study.

The time between the educational presentation (day one) and the virtual ROH (day four) was uniform. In contrast, the time between the educational presentation and the in-person ROH (between days eight and 11) varied by subject, possibly affecting performance. This varied time was a logistical necessity because we could only evaluate up to 19 in-person subjects per day due to the availability of the researchers and subjects. However, the difference among subjects was small and did not affect the overall results showing improved performance in hazard recognition following the virtual training.

Future considerations

Our study found that many subjects recorded some routine, non-hazardous items in the OR as hazards. Based on these misidentifications, we plan to incorporate additional education on routine and expected OR practices that are not hazardous [16,17].

While we instructed subjects to save their anonymized identifier for the duration of this study, future studies could more strongly stress the importance of subjects maintaining their identifier.

Due to the prohibitive costs and logistical challenges of closing a physical OR for all in-person simulation activities, we were unable to perform an in-person simulation for the control group to mirror the virtual ROH for the experimental group prior to the final in-person ROH scenario. Consequently, albeit virtual, the experimental group had additional exposure to the ROH compared to the control group. As such, while this study suggests that the virtual environment is effective and superior to didactic training alone, it cannot assess the superiority of the virtual training environment as compared to the in-person environment. Future studies may aim to assess whether the virtual ROH simulation is superior, inferior, or equivalent compared to in-person simulation. Nonetheless, the virtual ROH offers a viable and resource-efficient method for educating healthcare professionals, considering the significant challenges associated with closing a functional operating room for training purposes.

## Conclusions

This study highlights the effectiveness of virtual simulations in teaching pre-clinical medical students to recognize latent hazards in the OR, underscoring the value of integrating such innovative methodologies into medical curricula. The virtual ROH activity we developed to teach medical students about latent hazards in the OR successfully improved hazard recognition and received positive feedback from subjects regarding implementation into the medical student curriculum. It is easily accessible, convenient, and freely available for use in the curricula by other institutions. Alternatively, given the relatively low cost of producing online videos, medical schools or hospitals could develop their own teaching programs based on our described methodology. Our findings encourage further exploration into the development and application of virtual simulations across medical and healthcare training domains training, aiming to equip future professionals with the skills necessary for the complexities of clinical practice.

## Appendices

Appendix A

**Table 4:** Virtual operating room of hazards list (n = 52) DOB: date of birth; EKG: electrocardiogram; ET: endotracheal tube; IV: intravenous; MRN: medical record number; OR: operating room; SCD: sequential compression device.

Area/category	Hazard
Patient	The patient is not secured by the OR bed safety strap
	ET tube loosely taped
	The patient does not have a warming blanket
	The Foley bag is overfull
	Blood-tinged urine
	A catheter is over the leg
Anesthesiologist	Glove with a big hole
	Polo shirt/long sleeves under scrubs
	Hair out of bouffant
	Wearing earbuds
	Mask not properly worn (under ears)
Medications	Branded name of Zemuron instead of the generic name (rocuronium)
	No date and/or time recorded for Zemuron
	No date and/or time and dose unclear for propofol
	No date and/or time recorded for fentanyl
	No concentration was recorded for lidocaine
	Phenylephrine - trailing zero and/or dose is unclear
	Full syringe but no label
	Non-specific syringe issues - missing information/unclear
	Blood has no expiration date
	Ceftriaxone IV and the patient is allergic to ceftriaxone
	No concentration was recorded for Rocephin (ceftriaxone)
	Use of brand name Rocephin instead of the generic name (ceftriaxone)
	Rocephin's expiration date has passed
	IV bag has expired
	IV bag: the label was removed, and there is no concentration specified
	The blood type hanging is B+, but the patient is A+
Communication	The patient's last name "Yueng" is incorrect
	Whiteboard is undated
	The whiteboard was not fully visible due to an open door
	DOB discrepancies
	The right thigh marked instead of the abdomen
	Old patient sticker on the gown
	No allergy wristband
	No MRN number on the patient's wristband
Environment and supplies	Wrinkled Bovie pad
	Regular trash found in biohazard bin
	Regular trash discovered in sharps bin
	The biohazardous waste bin is overfull
	Expired Stericycle
	Only small-size gloves available
	OR door propped open
	Unplugged cords and/or tripping hazard
	EKG is disconnected
	Bair hugger and/or SCD not connected
	Tangled IV lines and monitor lines
	Yankauer found on the floor
	Two clocks in the room are inconsistent
	Phone wrapped in tape
	Uncapped sharps on circulator desk
	Open soda can
	Foley is placed on the sterile side of the equipment

Appendix B

**Table 5:** In-person operating room of hazards list (n = 39) DOB: date of birth; EKG: electrocardiogram; ET: endotracheal tube; IV: intravenous; MRN: medical record number; OR: operating room; SCD: sequential compression device.

Area/category	Hazard
Patient	No allergy wristband
	Discrepancy between allergy in chart and board
	Iodine out for prep when the patient is allergic
	Arm hanging off the table
	The patient is not secured with a safety belt
Anesthesiologist	Hair is out of bouffant
	The mask is worn below the nose
	The anesthesiologist is on the phone, not paying attention
	Not wearing gloves
Medications	Use of branded name (“Versed”) instead of the generic name (midazolam)
	No concentration for fentanyl
	Trailing zero - phenylephrine 200.0
	No ceftriaxone concentration
	No ceftriaxone date/time
Communication	Wrong date on the whiteboard
	No surgical marking
	Whiteboard without member names
	No procedure on the whiteboard
	DOB discrepancy with whiteboard/chart/or wristband
	Last name discrepancy with whiteboard/chart or wristband
Environment and supplies	Water on the floor with grey cord
	Loud music
	Granola bar on anesthesia table
	Foley is away from the table
	The clock time is incorrect
	Cord tripping hazard
	IV line under the stand
	Monitor lines are tangled
	The sharps container is expired
	The fire extinguisher is expired
	Stericycle is expired
	Only large-size gloves are available
	Electrosurgical unit touching sterile blue drape
	The phone is off the hook
	The OR light is placed too low, posing a risk of head injury
	The Purell dispenser is empty
	Bloody gloves in clear lined trash bin rather than red bag
	Sharp in the trash bin
	Foley is placed on the sterile side of the equipment

Appendix C

**Table 6:** Mistakenly identified hazards from the in-person room of hazards (n = 42) HIPAA: Health Insurance Portability and Accountability Act; IV: intravenous.

Area/category	Mistaken hazards
Patient	Fall risk bracelet over IV line
	Eyes taped shut
	Pulse oximeter on the wrong finger
	BP cable under the patient's head
	The patient is not wearing a mask (it's hanging)
	Bed too high with no support
Anesthesiologist	Anesthesiologist's feet on the table base
	The anesthesiologist's jacket is not buttoned
	The anesthesiologist is not wearing sterile clothing
Medications	The medication machine is not on
	No blood in the room
	Meds in the wrong units (mg instead of mc)
	Sodium chloride bag on anesthesia table
	Unlabeled IV bag
Communication	The patient chart is not on the computer
	The chart is out (HIPAA violation)
	Fall risk bracelet but not on chart
Environment and supplies	Open sterile equipment
	The Bovie machine is not turned on/plugged in
	Unorganized cords/not taped to the floor
	No handles on lights
	Foley on the ground/attached to trash but do not specify tripping hazard
	Sterile field not identified/patient not draped/patient exposed
	Sterile table far from the patient
	Bair Hugger not on
	Circulator computer off
	Drawer open/disorganized/not labeled
	The speaker should not be in the room
	Cabinets with low stock
	Gloves hanging out of the trash but do not mention bloody/not specific enough
	The bunny suit is too long
	Fire extinguisher blocked by trash
	Stylet and suction open but in wrapper
	Surgical instruments unorganized
	A drop of urine on the floor
	The table with iodine is open/not blue w/ sterile gloves on it
	Sharps/biohazard containers not easily accessible
	The area is crowded/unorganized in the corner with unnecessary items
	Tight spaces blocked by equipment/difficult to maneuver
	The trash can is right next to the door
	Syringes lying on the table for someone to take
	Biohazardous bin without a top
	IV touching floor
	The small trash can by the table is not marked or in the way
	Tools on the scrub table facing the patient
	Tray on top of biohazard bin
	Cotton swab in sterile iodine
	Trash in the sterile tray (it was the tags indicating sterility)
